# A Mathematical Model of Cancer Stem Cell Driven Tumor Initiation: Implications of Niche Size and Loss of Homeostatic Regulatory Mechanisms

**DOI:** 10.1371/journal.pone.0071128

**Published:** 2013-08-19

**Authors:** Sara N. Gentry, Trachette L. Jackson

**Affiliations:** 1 Department of Mathematics, University of Michigan, Ann Arbor, Michigan, United States of America; 2 Department of Mathematics, University of Michigan, Ann Arbor, Michigan, United States of America; National Cancer Institute, United States of America

## Abstract

Hierarchical organized tissue structures, with stem cell driven cell differentiation, are critical to the homeostatic maintenance of most tissues, and this underlying cellular architecture is potentially a critical player in the development of a many cancers. Here, we develop a mathematical model of mutation acquisition to investigate how deregulation of the mechanisms preserving stem cell homeostasis contributes to tumor initiation. A novel feature of the model is the inclusion of both extrinsic and intrinsic chemical signaling and interaction with the niche to control stem cell self-renewal. We use the model to simulate the effects of a variety of types and sequences of mutations and then compare and contrast all mutation pathways in order to determine which ones generate cancer cells fastest. The model predicts that the sequence in which mutations occur significantly affects the pace of tumorigenesis. In addition, tumor composition varies for different mutation pathways, so that some sequences generate tumors that are dominated by cancerous cells with all possible mutations, while others are primarily comprised of cells that more closely resemble normal cells with only one or two mutations. We are also able to show that, under certain circumstances, healthy stem cells diminish due to the displacement by mutated cells that have a competitive advantage in the niche. Finally, in the event that all homeostatic regulation is lost, exponential growth of the cancer population occurs in addition to the depletion of normal cells. This model helps to advance our understanding of how mutation acquisition affects mechanisms that influence cell-fate decisions and leads to the initiation of cancers.

## Introduction

**Figure 1:**
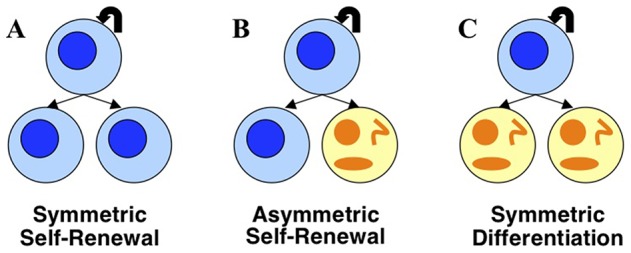
Stem cells are capable of three kinds of division. Stem cells may symmetrically self-renew to form two daughter stem cells (A), asymmetrically self-renew to form one stem cell and one progenitor cell (B), or symmetrically differentiate to form two progenitor cells (C).

All human tissues and organs are composed of a heterogeneous mix of cells, and not all cells are created equally in terms of their stage of development and their potential for proliferation and/or differentiation [1], [2]. Small populations of somatic stem cells, which sit at the top of the tissue hierarchy and play a critical role in tissue maintenance and repair, have been found in the brain, bone marrow, blood vessels, skeletal muscle, skin, teeth, heart, gut, liver, and other (although not all) organs and tissues [3]. These cells are characterized by their ability to self-renew, or make more stem cells, and their ability to produce progenitor cells that differentiate, ultimately generating all the cell types of the organ from which they originate [1], [4]. In adult tissues, an intricate balance exists between stem cell self-renewal and the generation of differentiated offspring [5]. One strategy by which stem cells can accomplish these two tasks, and maintain tissue homeostasis, is asymmetric cell division, whereby each stem cell divides to generate one daughter that retains stem cell properties and one daughter that differentiates into a progenitor cell [5], [4], [6]. Stem cells can also use symmetric divisions to self-renew and to generate differentiated progeny. Symmetric divisions are defined as the generation of daughter cells that are destined to acquire the same fate [4]. That is, stem cells can also divide to produce only stem-cell daughters (symmetric self-renewal) in some divisions and only differentiated daughters or progenitor cells (symmetric differentiation) in others. In principle, stem cells can rely either completely on symmetric divisions or on a combination of symmetric and asymmetric divisions, and the balance between these two modes is controlled by microenvironmental signals to produce appropriate numbers of stem cells and differentiated daughters [5], [4], [6]. These three different types of cell division are pictured in Figure 1.

The proliferation of stem cells is a tightly regulated, yet responsive, process, controlled by various mechanisms that are not fully understood. For instance, certain chemical signals may promote stem cell self renewal, while others initiate differentiation in response to a need for additional mature cells [4], [7]. Furthermore, environmental cues also influence stem-cell division [7]. Changes in the microenvironment have the ability to alter stem cell function and in some cases, could lead to malignancy, so it is important to understand how interactions within the surrounding microenvironment affect stem cells [8].

### The Stem-Cell Niche

Because the percentage of stem cells in healthy tissues is very small, these cells must be protected and maintained through tight regulation. It is believed that the stem cell niche is crucial in both aspects [9], [10], [11]. The niche can be thought of as the a restricted region in an organ that supports the self renewal divisions of stem cells. The niche is composed of both localized signaling cells and an extracellular matrix that controls stem-cell fate [5], [11]. One of the obstacles in stem cell research is the inability to scientifically reconstruct niches, which makes it difficult to maintain stem cells in vitro because signals from the niche affect stem-cell survival, self-renewal, and differentiation [9], [10], [11].

Within hierarchically structured tissues, if stem cells to do not self-renew, they differentiate into early progenitor cells that are responsible for expanding in number and eventually differentiating into fully mature cells that carry out specific functions for the tissue. Chemical signaling may influence the proliferation and differentiation of stem cells into different progeny types as demanded. Taking as an example the hematopoetic system, several colony-stimulating factors have been identified that impact stem and progenitor cell behavior. Interleukin-3 (IL-3) has been used as part of stem cell mobilization regimens and promotes the survival and proliferation of hematopoetic progenitors to increase production of various differentiated progeny including macrophages, granulocytes, mast cells, megakaryocytes, and erythrocytes [12], [13]. Macrophage colony-stimulating factor, M-CSF, and granulocyte colony-stimulating factor, G-CSF, promote survival, proliferation, and differentiation of mature and precursor macrophages and granulocytes, respectively [12], [14].

The stem cell niche also includes cytokines that are found in the microenvironment. Several proteins are associated with stem cell maintenance and differentiation, and scientists have recently begun identifying these molecules and their functions. For instance, the expression of Notch, a transmembrane protein used in cell-to-cell communication, may promote stem-cell quiescence, and integrins may affect the interactions between stem cells and the extracellular matrix [9]. The growth-promoting Wnt family of proteins are prevalent during embryogenesis and may play a role in cell proliferation and differentiation [9]. Independence from the control of niche signaling leads to cancer, which is further evidence that the niche is crucial in maintaining tissue balance. Loss of tumor suppressor Pten causes HSC mobilization and leukemia [15]. Alteration in the balance between the anti-growth bone morphongenic protein, BMP, and Wnt signaling promotes tumorigensis [16], [15]. Therefore, it is clear that signaling pathways in the niche mediate tissue homeostasis.

### Cancer Stem Cells

The realization that cancers may rely on tumor-initiating cells that share many features of normal stem cells has changed the perspective on the origins of and treatment strategies for the neoplastic disease. Cancer stem cells (CSCs) have been defined as cells within a tumor that possess the capacity to self-renew and to cause the heterogeneous lineages of cancer cells that comprise the tumor [17]. The cancer stem cell hypothesis suggests that malignant growth is driven by a subpopulation of stem-like, tumor-initiating cells [18] and that these cells are responsible for tumor growth, resistance, and recurrence [19]. These cancer stem cells may arise from mutations in stem cells or from early progenitors that have acquired stem-cell characteristics [20]. Cancer stem cells have now been identified in tumors of the breast, brain, colon, and blood, among others [21], [22], [23], [24], [25], [19]. By maintaining at least some of the properties of their tissue of origin, CSCs give rise to tumors that phenotypically resemble their origin, either by morphology or by expression of tissue-specific genes. However, what distinguishes cancerous tissue from normal tissue is the loss of homeostatic mechanisms that maintain normal cell numbers, and much of this regulation normally occurs at the stem cell level [26].

In addition to providing an elegant model for carcinogenesis, the CSC hypothesis raises several important experimental and clinical implications. First, if a population of biologically unique CSCs exists, then tumor cells lacking stem cell properties will not be able to initiate self-propagating tumors, regardless of their differentiation status or proliferative capacity [26]. Furthermore, the existence of CSC has the clinical implication that curative therapy will require complete elimination of the CSC population. Patients who show an initial response to treatment may ultimately relapse if even a small number of CSCs survive. On the other hand, targeted therapies that eliminate the CSC population offer the potential for cure. Given this promise, it is not surprising that the CSC hypothesis has attracted so much attention in recent years [26].

Due to the difficulty of isolating and studying stem cells experimentally, mathematical modeling can provide further insight into the growth dynamics involved during tumorigenesis in hierarchical tissue and can shed light on the potential of stem-cell targeted treatments.

### Previous Mathematical Models

For decades, mathematical models have been employed to help address some of the most pressing questions associated with tumor growth. For example, it is well known that cancer is a multi-step process in which somatic mutations accumulate to initiate malignancy [27], [28] and mathematical modeling has provided insightful information regarding these mutagenic pathways. In particular, modeling has helped to highlight which mutations most increase cell fitness, determine which mutations speed cancer onset and progression, and predict the order of mutation acquisition in specific malignancies. Existing models of cancer stem cell driven tumor growth have utilized discrete [29] approaches; ordinary [30], [31], [32], [33], [34], [35], [36], [37], [38] and partial [39], [40] differential equations; and hybrid-cellular automaton models [41], [42]. Most of these models focus on the growth dynamics and the implications of treating tumors with phenotypically different, hierarchically structured cellular populations, instead of focusing on the mutation pathways that lead to this heterogeneous mixture of cells. The order of mutation acquisition likely affects the tempo of malignant growth and Spencer et al. developed a mathematical model, consisting of a system of ODEs to investigate which pathway instigated the fastest tumor growth [31]. Loosely based on breast cancer data, their model predicted that evasion of apoptosis, followed by increased replication, then angiogenesis, then genetic instability constituted the fastest path to cancer. Their model, however, did not actually track the sequential order in which mutations were acquired. For instance, cells with both the ability to evade apoptosis and to increase their proliferative potential were collectively combined into the same subpopulation and the historical order of mutation was not distinguished. Since cells with the mutation for evasion of apoptosis surpassed all other populations expressing only one mutation, it was assumed that this was the first event in the fastest sequence. Similar calculations concluded subsequent transformations to establish the fastest path, ignoring the specific order of mutagenic events.

To specifically model mutli-step tumorigenesis in the breast, Enderling et al. developed a model that incorporated the multi-step approach of Spencer et al. for a specific mutation sequence [34]. Breast stem cells sequentially acquired two mutations to knock out one tumor suppressor gene, followed by an additional two mutations that removed a second tumor suppressor gene. Cells that had completely lost both tumor suppressor gene alleles were considered cancerous stem cells. In addition, the authors included radially symmetric spatial aspects to simulate tumor growth. As a result, non-cancerous cell populations were modeled with ordinary differential equations, while cancerous cells were modeled with a partial differential equation that was dependent both on time and a one-dimensional space variable. This model predicted that in order to generate a tumor within the clinically observed time, either mutations are acquired before puberty that make cells predisposed to accumulating additional mutations or genetic instability occurs early.

Several models have been developed that emphasize the significance of genetic instability in cancer-initiation. These models demonstrate that genetic instability promotes faster tumor growth. Beckman and Loeb used a deterministic model to figure out the probability that a cell would become cancerous based on the order in which the mutator phenotype was acquired [35]. They concluded that genetic instability confers the greatest advantage when it occurs as the initial mutagenic event and becomes increasingly significant in highly proliferative tissues. Their results confirm those of Michor et al., who established that chromosomal instability is likely an early event in the initiation of colon cancer [32], [33].

To demonstrate the competitive advantage of mutator cells over those that are not unstable, Komarova and Wodarz developed a differential equations model that contrasted the fitness of these two cell types [30]. This study hypothesized that mutator cells do not necessarily expand faster than stable cells during mutation acquisition. For instance, the magnitude of mutation rate and the extent to which apoptotic checkpoints remain intact both influence whether the mutator or stable phenotype is favored. Therefore, they conclude genetic instability is most tumorigenic when programmed cell death is previously deregulated.

Clearly the order of mutation acquisition affects tumor dynamics. The multi-step models mentioned here did not consider how hierarchical organization may affect the pathways that lead to tumorigenesis. Due to the longevity and increased proliferative potential of stem cells in comparison to terminally differentiated cells, it is reasonable to propose that transformed stem cells are more capable of propagating malignancy. As a result, segregating stem, progenitor, and differentiated cells in multi-step models can generate more accurate results of mutation acquisition in hierarchical tissue.

Using a simple discrete mathematical model, Tomlinson and Bodmer established that mutations at the stem cell level were most significant in promoting malignancy [29]. They argued that expansion could result from the failure of apoptosis or the block of differentiation rather than unbridled proliferation. Furthermore, they were able to demonstrate the importance of incorporating tissue hierarchy in cancer models, because mutated progenitors and differentiated cells were unable to cause exponential growth, unlike mutated stem cells. The model was first developed to simulate a normal system in homeostasis. Predictions of cancer cells were made based on the variation of model parameters, so the actual process of mutation acquisition was not studied.

To model the cancer stem cell hypothesis in neural tumors, Ganguly and Puri created a deterministic model of tumorigenesis that compartmentalized stem, progenitor, and differentiated cells as well as their mutated counterparts [43]. In this model, stem and progenitor cells could become cancer cells through the acquisition of one mutation, so the multi-step pathways initiating cancer were not explicitly explored. The model predicted that mutations occurring in stem cells had more of an effect on tumorigenesis than mutations to progenitors. In addition, by incorporating feedback regulatory mechanisms between the various cell populations, it was suggested that repeated injury to mature cells, such as repeated radiation, could promote stem-cell proliferation, which in turn could increase mutation acquisition. This model is a good example of tumorigenesis in regulated hierarchical tissue and emphasizes the impact of mutations in stem cells, but it does not investigate the sequential order of mutation acquisition that promotes cancer.

In order to simulate the cancer stem cell hypothesis mathematically, it is necessary to model cancer stem cells as a distinct subpopulation from other tumor cells. Furthermore, tissue hierarchy must be considered because stem, progenitor, and differentiated cells have very different properties. In this paper, we develop a mathematical model of mutation acquisition in a normal, hierarchical tissue and use the model to investigate how deregulation of the mechanisms preserving tissue homeostasis contributes to cancer. For each mutation pathway considered, we define the onset of malignancy as the time at which the first cancer stem cell is formed, and this is in turn used to define the fastest pathway to tumorigenesis. Importantly,the model predicts that the order in which mutations are acquired significantly impacts tumor composition and dictates the pace of tumor formation.

## Materials and Methods

Just as hierarchical structure influences the multi-step process of tumorigenesis, mechanisms governing tissue homeostasis may also significantly impact cancer growth dynamics. In order to investigate the sequential acquisition of mutations that initiate cancer in a hierarchical tissue under hemostatic regulation, we develop a mathematical model that tracks normal and mutated stem and differentiated cells.

### Model Development

**Figure 2:**
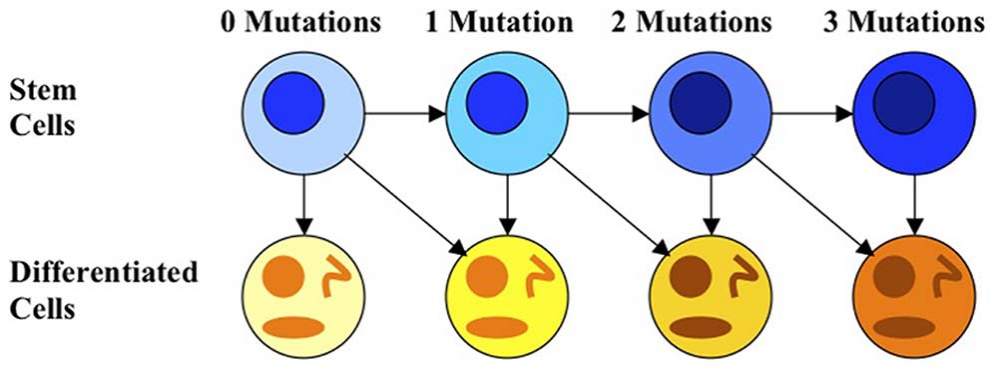
Mutation acquisition in stem cells and the formation of abnormal progeny. Stem cells acquire mutations with small probability during each division and pass on mutations to their progeny. Terminally differentiated cells are fully mature, and therefore, do not divide and acquire additional mutations.

We consider three classes of point mutations; namely, those that deregulate proliferation, result in evasion of apoptosis, and enhance genetic instability. These types of mutations are likely involved in the early stages of cancer, whereas mutations causing angiogenesis and metastasis are likely acquired in later stages, after a tumor has grown beyond a certain threshold size [28]. The mathematical model, therefore, consists of eight ordinary differential equations representing stem and differentiated cells with mutations in 0,1,2, or 3 of the classes described above, 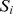 and 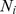; respectively. Normal stem cells, 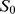, acquire their first mutation at rate 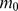, at which time they become 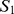 cells. Likewise, 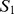 cells acquire the second mutation at rate 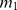 to become 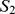 cells, and 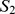 cells acquire the third mutation at rate 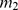 to become 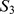 cells. Stem cells are the source for differentiated cells through asymmetric and symmetric differentiation divisions. Intermediate populations of progenitors are not modeled explicitly. Instead, their presence is accounted for via an amplification factor, 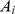, which incorporates the average number of progeny resulting from the differentiation of a precursor cell with 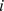 mutations as well as the rate of division of these cells. Terminally differentiated cells cannot complete further divisions nor can they mutate; therefore stem cells are the only cells that can acquire additional mutations. A schematic of the flow of cells from one population to another is shown in Figure 2.

Stem cells in each population class (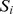) proliferate at rate 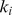. We further assume that each stem cell encounters one of four fates during each division: symmetric self-renewal, asymmetric self-renewal, symmetric commitment differentiation, and apoptosis. Stem cells symmetrically self-renew with probability 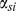, which increases the stem cell pool by one. Stem cells asymetrically self-renew with probability 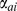, which does not change the stem cell pool, but increases the progenitor pool of population class 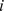 by one. Stem cells symmetrically differentiate with probability 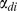, which decreases the stem cell pool by one and increases the progenitor pool of population class 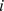 by two. Finally, stem cells die with probability 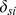, and it therefore follows that 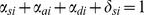. It is assumed in this model that stem cells are only marked for death or differentiation as a result of dividing, though the model equations could easily be slightly modified to allow for division-independent differentiation and apoptosis. The mathematical model equations are described in the subsections that follow.

### Stem Cell Equations

We begin by deriving equations for the stem cell pool, which includes cells that have 0,1,2, or 3 mutations.
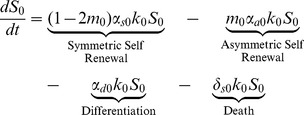
(1)


The first term in (1), which describes symmetric self-renewal, incorporates both the increase in population due to symmetric self-renewal (that is, 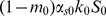) and the decrease in the population due to mutations that occur during symmetric self-renewal (namely, 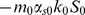). Note that if 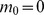, implying that mutations are not possible, the stem cell pool increases by one with probability 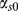 due to symmetric self-renewal and decreases by one with probability 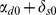 due to differentiation and death; respectively. Where as if 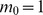 so that mutations are guaranteed with every division, the stem cell pool will inevitably decrease.
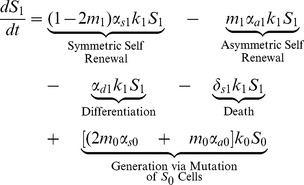
(2)

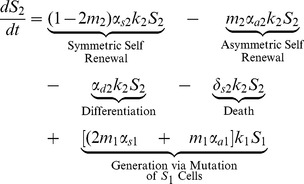
(3)


The first four terms in equations (2) and (3) for stem cells with one or two mutations are analogous to those in equation (1). The last two terms describe the generation of 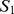 and 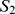 as a result of mutations that have occurred in the 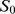 and 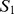 populations; respectively.
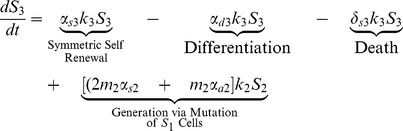
(4)


Finally, stem cells that have acquired all three mutations are generated by mutations that have occurred in the 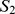 population. These cells can then only self-renew (symmetrically or asymetrically), differentiate, or die.

### Differentiated Cell Equations

Stem cells, both normal and mutated, give rise to differentiated cells with those same properties. Terminally differentiated cells cannot divide or mutate; they are simply generated by stem cells and once they have lived their natural lifespan, they die. The equation for the population of differentiated cells with no mutations is given in (5).
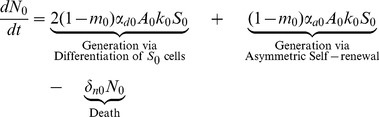
(5)


The first two terms in equation (5) represents the generation of normal terminally differentiated cells through both differentiation and asymmetric self-renewal of normal stem cells and the third term describes natural cell death.
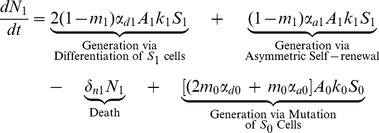
(6)

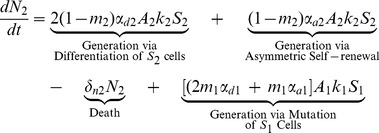
(7)


The first three terms in equations (6) and (7) for differentiated cells with one or two mutations are analogous to those in equation (5). The last two terms describe the generation of 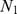 and 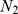 as a result of mutations that have occurred in the 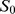 and 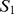 populations; respectively.
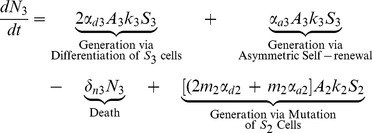
(8)


Finally, terminally differentiated cells with all three mutations are generated by i) differentiation and asymmetric self-renewal of stem cells with all three mutations and ii) mutations that have occurred in the 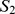 population. Again, in equations (5)–(8), 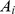, is the amplification factor that incorporates the average number of progeny resulting from the differentiation of a precursor cell with 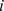 mutations as well as the rate of division of these cells. It is assumed that cells may only acquire one mutation at a time. Cells with 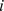 mutations may alter any of the model parameters, depending on which mutation is acquired, thus each parameter is denoted with an i-subscript to allow these values to differ from the baseline value.

All simulations start with 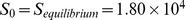 and 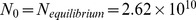, which were determined by running the model to steady state from the starting point of a single non-mutated stem cell. All mutated cell populations (for stem and differentiated) start at zero.

### Probabilities of Stem Cell Division

We assume that the probabilities for stem cell division are not constant, but rather are regulated by chemical signaling and environmental (niche) constraints [44], [45], [5], [4]. We further assume that the probabilities for stem cell division have the same functional form for all stem cell populations; therefore, when mutations occur, the functional forms do not change, only the parameters in them are varied.
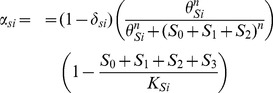
(9)

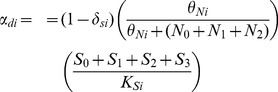
(10)

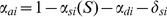
(11)


Experimental evidence shows that symmetric self-renewal of stem cells may be controlled by both extrinsic and intrinsic chemical signaling [44], [45], [5], [4]. Certain environmental cues may promote self-renewal, while others promote differentiation. Similarly, proteins produced within the cell can affect how a stem cell divides in an autocrine manner. The Hill function in the 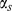 equation is used to describe the effect of chemical signals on the probability of symmetric self-renewal. This functional form has been used in previous mathematical models of hematopoiesis and can be derived from receptor ligand binding kinetics [46], [47]. It is assumed that cancer cells with all three mutations (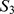) do not produce signals to inhibit symmetric self-renewal, and thus they are omitted from the Hill function in 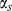. As the number of stem cells that produce chemical signals for self-renewal, 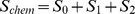, approaches zero, the probability of symmetric self-renewal based on chemical signaling approaches the maximum value of one. The parameter 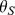 may be interpreted as the number of stem cells for which the probability of symmetric self-renewal resulting from chemical signaling is equal to one half. Higher values of the exponent 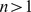 increase the sensitivity of stem cells to the chemical signals for symmetric self-renewal.

**Figure 3:**
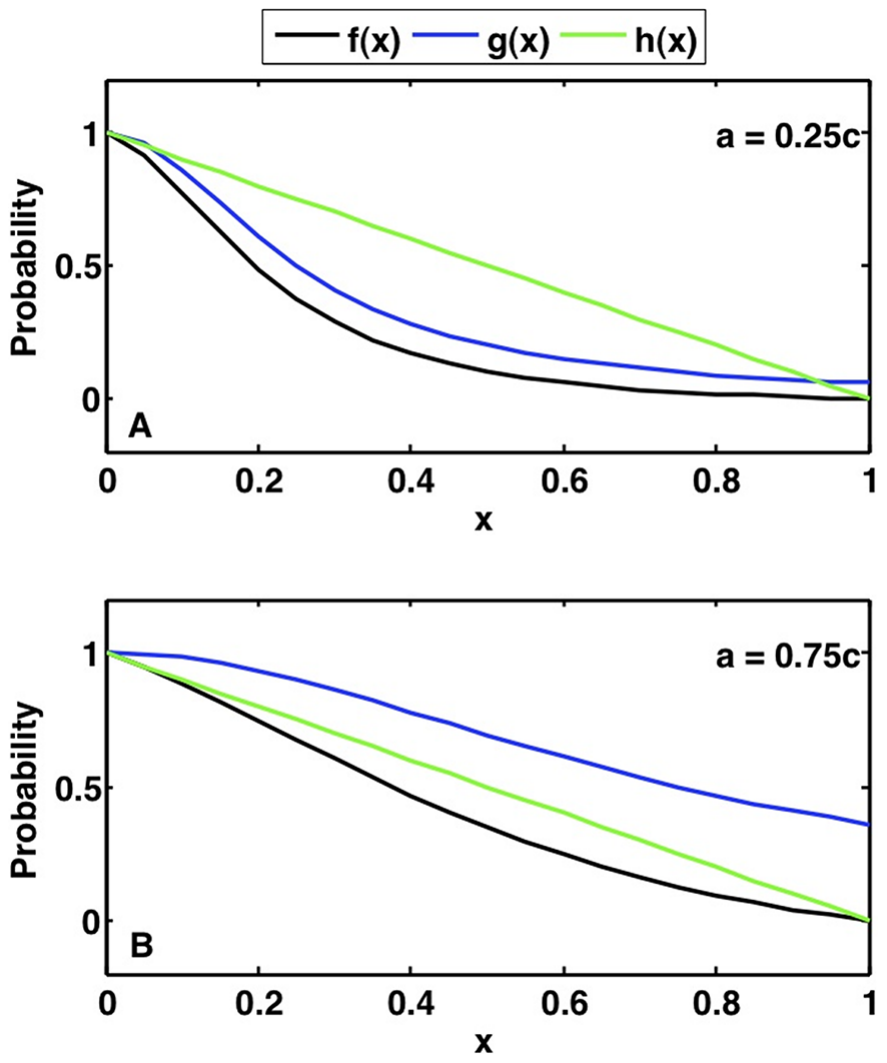
Functional forms used to determine the probability of symmetric self-renewal. The probability of symmetric self-renewal follows that of function 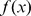, which takes into account both chemical interactions and niche control. Probability based solely on chemical signaling is given by function 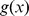, and probability based solely on niche control is given by function 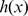.

Stem cell interaction with the niche is also necessary for maintaining stemness, thus the logistic term in 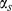 captures the physical restraint of the niche size on symmetric self-renewal due to limited available space for stem-cell sustenance [48]. Cancer cells do take up space within the stem cell niche, and as a result, cancer stem cells are incorporated in the logistic term. Note that symmetric self-renewal cannot exceed 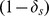, since it is assumed that stem cells die with constant probability. To further illustrate why both chemical signaling and niche control are found in the functional form for symmetric self-renewal, consider the function 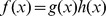, where 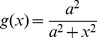 and 
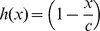
, with 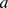 and 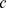 being arbitrary positive constants. Note that our choice for 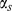 resembles 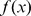 in that the chemical signaling function shares the form of g(x), and the niche control function is similar to 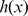. Figure 3 plots 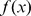 for two different values of the parameter, 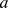. In Figure 3A, symmetric self-renewal is more restricted by chemical signaling than by the niche, whereas the converse is true in Figure 3B. In both cases, 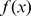 captures key components of both the Hill function and the logistic function. Note that 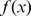 is zero when the niche is full, which occurs when 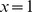 in this example due to the logistic term, but 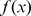 also has the qualitative behavior, including the changes in concavity, associated with the Hill function due to chemical signaling. As a result, both functional forms are incorporated into 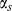 in order to capture both traits.

The Hill function in 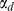 reflects the effects of chemical signaling that promote or suppress differentiation depending on the existing population of mature cells and has been used in previous models of cyclical neutropenia and periodic chronic myelogenous leukemia [47], [49]. Note that cancer differentiated cells 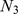 are no longer signaling properly and do not influence symmetric differentiation. In addition, the term 
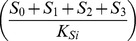
 ensures preference is given to self-renewal over differentiation in cases where both stem and differentiated cells are depleted, so the system is not compromised or extinguished in such cases [50] In the equation for 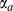, it is important to note that the model does not discriminate based on the mechanism by which asymmetric division is achieved. The asymmetric division term can encompass divisions that occur by the immortal-strand hypothesis or divisions in which two identical cells determine their fates from cues after division. The stem-cell division types are merely classified by the state of the two daughter cells at the time of their subsequent division.

We assume that the probabilities for stem cell division have the same form for all stem cell populations. However, the parameter 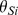 is altered when a mutation occurs that affects the symmetric self-renewal response associated with chemical signaling and 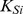 is varied when a mutation occurs that affects a stem cell's dependency on the niche.

### Parameter Values

The parameter values for both normal and mutated cells are presented in Table 1. Table S1 and Table S2 explicitly list the parameters used for three of the six pathways for each of the types of the R mutations considered. The remaining pathway parameters can be easily determined by permuting these appropriately for the desired pathway. Although values are derived from the hematopoietic system, the model can be easily applied to other tissues by using appropriate parameter values.

**Table 1:** Model Parameters.

Units	Parameter	Description	Normal Value	Mutated Value
%	*μ*	% cycling stem cells	0.1528 [44], [60], [61] *	
per day	*r*	proliferation rate of cycling cells	0.2310 [[61], [62] *	
per day	*k* = *μr*	proliferation rate of stem cells	0.035	2*k*
per day	*δ_S_*	death rate of stem cells	0.05	0.5*δ_S_*
	*m*	probability of mutation	10^−6^	10^−4^
per day	*δ_N_*	death rate of differentiated cells	2.4	0.5*δ_N_*
	*A_i_*, *i* = 0,1,2,3	amplification factor	1.1×10^8^	
# cells	*θ_S_*	half-saturation constant	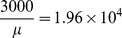	2*θ_S_*
# cells	*K_S_*	niche carrying capacity	3×10^4^	2*K_S_*
# cells	*θ_N_*	half-saturation constant	1×^10^	
	*n*	Hill coefficient	2	

* Our values are chosen from the ranges found in the references listed.

Although hematopoietic stem cells are better understood than stem cells in other tissues, there is still much uncertainty concerning *in vivo* measurements. Part of the discrepancy comes from the process of isolating stem cells. There are several markers that isolate immature cells from those that are more differentiated, but it can be difficult to separate stem cells from early progenitor cells. Therefore, it is not uncommon for a population of stem cells to also include early progenitor cells, which can taint the true measurements of stem versus early progenitor cells. As a result, the current literature includes a wide range of values regarding hematopoietic stem-cell kinetics. Because this mathematical model separates stem cells from all other cells, the parameters used here attempt to reflect the most purified stem-cell population.

## Results

We define the *mutation pathway* as the order in which mutations occur and we compare and contrast all mutation pathways in order to determine which sequences generate cancer cells fastest. Several types of genetic transformations have been implicated in oncogenesis, but in this investigation, focus is directed towards somatic mutations that occur during DNA replication. To examine the initiation of cancer, three mutations are considered here. The D mutation decreases the percentage of stem cells that go through apoptosis and decreases the maximum death rate of non-stem cells. The G mutation increases the rate at which subsequent mutations are acquired. The R mutation alters cell proliferation, by either increasing the rate of proliferation or shifting the balance of stem-cell division to favor symmetric self-renewal (each of these cases will be considered separately). A cell is considered to be healthy and normal if it does not have any mutations and assumed to be cancerous once it has acquired all three mutations. For model simulations, all mutations are one-hit, though mutations requiring two genetic events could easily be incorporated simply by increasing the number of mutations that must occur to malignantly transform a cell. Mutations enabling angiogenesis and metastasis are not considered because they are considered to occur later in disease progression [28], [51].

The order in which mutations are acquired is noted by the order in which D, G, and R are listed. There are six possible sequences in which the mutations accumulate:
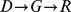


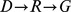


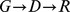


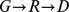


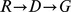


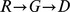



Tumor dynamics are compared and contrasted for all six pathways. Note that each pathway produces cancer cells that have acquired the same three mutations, but each pathway is different in the order in which mutations occur. Because a specific cancer is not being modeled, it is assumed that for each D, G, and R point mutation in the model, there are approximately 100 genes that may cause transformation [31]. As a result, the mutation rate is one hundred times the suggested mutation rate of 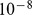 per division [33], [31]. Among genes mutated are those that function in guaranteeing the stability of the genome. Current experiments have centered on two mechanisms for the generation of genomic instability: mutations in mismatch repair genes (MMR) resulting in microsatellite instability and mutations in genes that are required for chromosomal segregation resulting in chromosomal aberrations [52]. We will assume the G-mutation is our model is the result of mutations in mismatch repair gene because this mechanism for genomic instability is associated with a marked increase in the mutation rate [53]. It has been established that MMR-deficient cells have a 100-fold to 1000-fold increase in their mutation rate [53].

Because cancer stem cells are believed to drive tumor growth, the emergence of the first cancer stem cell establishes the onset of malignancy in our model. As a result, the time required to generate the first cancer stem cell is recorded for each mutation pathway and we define the *fastest* pathway as the sequential order of mutations that leads to the quickest emergence of the first cancer stem cell. In the subsections below, we consider separately two key types of R mutations: Case 1 - mutations that increase rate of stem cell division without affecting the type of division (symmetric self-renewal, asymmetric self-renewal, differentiation); and Case 2 - mutations that increase probability of stem cell symmetric self-renewal without affecting the rate of division. For each of these types of R mutations, we consider three subcases: A) advantageous mutations - all mutations increase the cell's competitive advantage in some way; B) lethal mutations - mutations occurring in cells that have not yet acquired the ability to evade apoptosis are disadvantageous and increase cell death; and C) regulatory mutations - mutations leading to independence of niche signaling and the loss of feedback interactions that dictate the mode of stem-cell division. Finally we investigate the consequences of increased niche size for mutated cancer stem cells.

### Increased Stem Cell Proliferation

Cellular proliferation is increased in various forms of cancer. For instance, overexpression of the potassium channel TREK-1, the androgen receptor, and cyclin D1 have each been implicated in increased proliferation in prostate cancer cells [54], [55], [56]. It has also been suggested that BCR-ABL, which is expressed in CML patients, increases the rate at which hematopoietic cells divide [7]. Simulations of the model equations without mutations show that the stem cell proliferation rate is a key parameter in determining the rate of tissue generation. Increasing the stem cell proliferation rate alone has minimal effect on the homeostasis level of stem cells and as long as symmetric self-renewal and symmetric commitment differentiation are balanced, the the stem cell population reaches equilibrium. The number of differentiated progeny significantly increases because of the increased number of asymmetric self-renewal and symmetric commitment differentiation stem cell divisions, but also reaches equilibrium due to stem cell homeostasis.

When a mutation occurs that affects the cellular replication rate, the result is stem cells completing more divisions in a given amount of time, which provides a greater opportunity for cells to acquire additional mutations. Assuming that an R (replication) mutation doubles the stem cell proliferation rate, three sub-cases can be considered. In the first case, all mutations are advantageous and give the mutated cell added benefits over normal cells. In the second case, mutated cells that have acquired G and/or R mutations without a previous D mutation have increased cell death. In the third case, cells with G and/or R are penalized without the D mutation with the added assumption that cancer cells do not retain feedback regulatory mechanisms. Specifically in this case, the probabilities of stem cell division are constant in cells with all three mutations. For each of these sub-cases, the pathway that causes the fastest emergence of the cancer stem cell population is determined, and the change in tissue composition over time is discussed.

#### All Mutations are Advantageous

**Figure 4:**
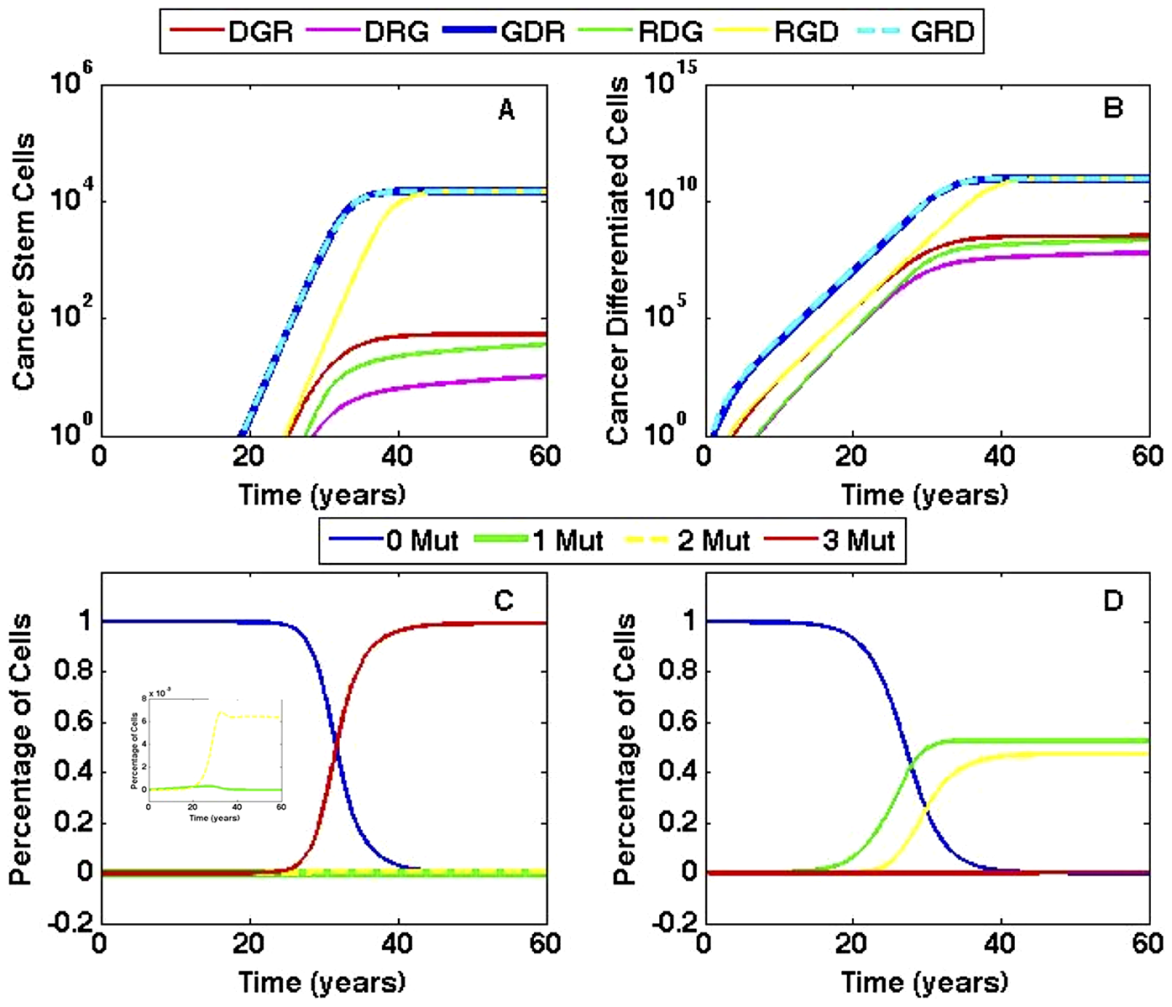
Comparison of pathways when all mutations are advantageous. The order in which the G mutation is acquired determines the fastest paths. Cells with the G mutation have probability of mutation 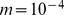; cells with the D mutation have stem cell death rate 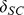 = 0.025/day and differentiated cell death rate 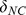 = 1.2/day; cells with the R mutation have stem cell proliferation rate 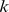 = 0.07/day. (A) Cancer stem cells formed in each pathway are plotted versus time. The GDR pathway has the first cancer stem cell, followed very closely by the GRD pathway. (B) Differentiated cancer cells are plotted versus time for each pathway. The growth of differentiated cancer cells mirrors the growth of cancer stem cells in each pathway. (C) Tissue composition for the fastest pathway, GDR, versus time. The inset provides an expanded view of the percentage of cells with one and two mutations. (D) Tissue composition for the slowest pathway, DRG, versus time.

Consider a case in which every mutation gives advantage to the cell. That is, each mutation increases the cells fitness and cell death does not increase in attempting to eliminate the mutated cell. Specifically, the D mutation decreases the probability of stem cell death by half and decreases the death rate of differentiating cells by half. The G mutation augments genetic instability, increasing the rate at which mutations are acquired from 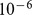 to 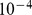. The R mutation doubles the proliferation rate of stem cells. Under these conditions, genetic instability is the most significant contributor to cancer onset (see Figure 4). Genetic instability predisposes the cells to accumulating additional mutations, which quickens the time in forming the first cancer stem cell. The GDR and GRD pathways are fastest, with the first cancer stem cell forming in nineteen years and the slowest pathway, DRG, is nearly nine years slower. Cancer stem cells and cancer differentiated cells of all pathways are plotted versus time in Figure 4A–B, respectively. This figure shows that there is negligible difference between the GDR and GRD pathways, however when the G mutation is acquired second or last, it significantly slow the pace of tumor initiation. Thus, it is evident that the order in which the G mutation is acquired determines the speed of cancer stem cell generation.

If feedback mechanisms are neglected, the system would not adjust to the increasing number of mutated cells in the tissue. Normal cells would remain in homeostasis, while all mutated populations would expand without bound. With the inclusion of feedback in the model, mutated cells do not grow exponentially for all time, but instead displace non-mutated cells, until healthy cells diminish from the system entirely. The tissue composition of the fastest pathway, GDR, is plotted in Figure 4C. Non-mutated cells dominate the tissue for approximately thirty years, after which cancer cells are the majority. In contrast, when following the slowest pathway, DRG, cells with only one and two mutations eventually take over the tissue and cancer cells remain a small percentage, as demonstrated in Figure 4D. Therefore, not only does the time to form the first cancer stem cell vary between various pathways, but the order in which mutations are acquired also determines the dominance of cancer cells within the tissue.

The fastest pathways leading to a cancer stem cell are those in which G is acquired first, while the slowest acquire G last. The significance of the G mutation may at first seem surprising because it does not increase the cells fitness as the D and R mutations do. In fact, the G mutation might be thought of as a silent mutation that does not appear to give the mutated cell any advantage. However, the acquisition of G accelerates the rate at which additional mutations are acquired, and therefore decreases the time required to generate the first cancer stem cell. Furthermore, the G mutation alone does not increase the potential steady state level for mutated cell populations; that is, cells with only a G mutation will not surpass the steady state of normal cells. Subsequent mutations will directly increase the cells fitness and increase the steady state that may be attained by cells with two and three mutations. In contrast, when G is acquired later, cells with only one mutation have increased fitness and a higher steady state potential, which causes the niche to fill with these cells. As a result, pathways in which G is acquired early will produce cancer populations with higher numbers than those in which G is acquired last, because the increased steady state level potential does not increase until cells have obtained additional mutations.

In this case, the sequential order of the G mutation is the most important in determining the fastest pathway, but no such conclusion can be made about the order of D and R mutations. Whether D or R occurs earlier in the fastest pathway depends on the amount of change between normal and mutated proliferation and death rates. For instance, using certain parameter values, GDR could be the fastest, while for others it would predict that GRD is fastest. Over a wide range of parameters, however, the impact of the G mutation is still most significant in determining the time to cancer onset. As a result, the conclusion that genetic instability dictates the time to malignancy is robust.

#### Lethal Mutations

Now a case is investigated in which every mutation does not give the cell added advantage, but can instead increase cell death as a result of cellular machinery recognizing the mutation and forcing the cell into apoptosis. The D, G, and R mutations still have the same definitions, but now it is assumed that cells that have acquired the G and/or R mutation without the D mutation have an increased rate of apoptosis such that the probability of stem cell death is 0.95 during division. For example, in the GRD pathway, cells with the G mutation only obtain the ability to mutate faster but they also have a higher death rate. Cells that are able to acquire the next mutation, R, have both genetic instability as well as increased proliferation, but the probability of cell death remains high since apoptosis is favored due to the recognition of mutation. Once D is acquired, then the cell has increased its ability to evade apoptosis, which lowers the death rate, and the advantages gained in the previous G and R mutations remain.

**Figure 5:**
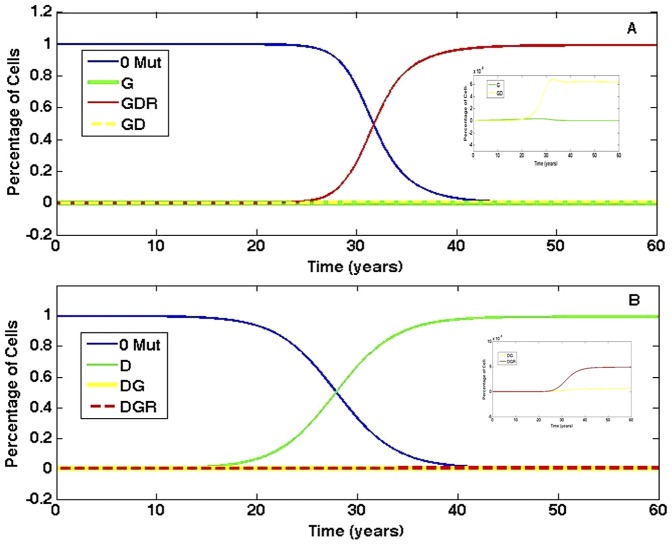
Comparison of tissue composition for fastest pathways when all mutations are advantageous versus when some are lethal. (A) The tissue composition of the fastest pathway, GDR, when all mutations are advantageous. Cells with the G mutation have probability of mutation 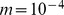; cells with the D mutation have stem cell death rate 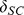 = 0.025/day and differentiated cell death rate 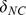 = 1.2/day; cells with the R mutation have stem cell proliferation rate 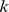 = 0.07/day. The majority of tissue is eventually comprised of cells with all three mutations. The inset provides an expanded view of the percentage of cells with one and two mutations. (B) The tissue composition of the fastest pathway, DGR, when some mutations are lethal. Its tissue composition is strikingly different in that the majority of cells eventually have only one mutation and cancer cells are a small percentage of the tissue. Parameter values are the same as those in (A). The inset provides an expanded view of the percentage of cells with two and three mutations.

Unlike the findings when all mutations are advantageous, the order in which genetic instability is acquired is not important in determining the pace in which cancer is initiated when lethal mutations are considered. To illustrate this conclusion, consider the DGR and RGD pathways. Both pathways acquire G second, but DGR is the fastest pathway while RGD is the slowest. Therefore, the significance of genetic instability is minimized when it is a lethal mutation. Instead, if the probability of cell death increases in mutated cells that cannot already evade apoptosis, then acquiring the D mutation first contributes to the fastest emergence of cancer stem cells. Once cells obtain the D mutation, then all subsequent mutations are advantageous, which is not true if either G or R is acquired first. Not only does the fastest pathway change under the assumption of advantageous versus lethal mutations, but the tissue compositions of the fastest pathways are contrasting. Figure 5 compares the tissue composition between the fastest pathway when all mutations are advantageous, GDR, and the fastest pathway under the assumption of lethal mutations, DGR. With the GDR pathway, cancer cells (cells with all three mutations) take over the tissue in thirty years. In stark contrast, cells with only one mutation, namely the D mutation, eventually dominate tissue following the DGR pathway. Therefore, it could be argued that the all-advantageous sequence GDR is a more aggressive form of disease as the tissue composition is furthest from its normal state. The initiation of cancer is delayed 10–20 years in pathways for which D is not first. Furthermore, the tissue composition for each pathway resembles the pathway's composition under the assumption that all mutations are advantageous, but the dominance of mutated subpopulations is delayed. For example, in the pathway GDR in which G is lethal without D, cancer cells still eventually dominate the tissue, though it is ten years later than when all mutations are advantageous. The prevalence of cancer cells in the tissue implies that this pathway simulates a disease that progresses quickly, even though it takes longer to initiate than the DGR pathway. In contrast, the DGR pathway produces a tumor that primarily consists of cells with one mutation. The D mutation is the only mutation that can directly increase the steady state of stem cells, while both the G and R mutations affect how quickly cells move from one mutated population to the next. As a result, when D is acquired first, stem cells with one mutation outnumber normal cells, and this advantage for expansion allows the clone with the D mutation to dominate the tissue.

In the event that mutations are lethal without a previously acquired ability to evade apoptosis, the fastest pathway begins with the D mutation. The two cases explored thus far demonstrate that unrestricted growth is not possible in tissues that maintain some level of regulation. Hypercellularity can occur, but some level of equilibrium is achieved, even if it is abnormal. The next section will consider the effects on tumor dynamics when regulatory mechanisms are removed in cancerous cells.

#### Mutations Affecting Regulatory Mechanisms

We have explored, thus far, mutations that affect death, genetic instability, and proliferation of stem cells; however, the regulatory mechanisms governing stem cell division pattern have remained intact. Because cancer cells can become self-sufficient in growth signals, it is likely that cancer cells could escape control from regulatory mechanisms [28], [51]. To investigate this possibility, begin with the case of lethal G and R mutations in the absence of D, and further assume that cells that have acquired both the D and R mutations become independent of niche signaling and lose feedback interactions that dictate the mode of stem cell division. In other words, the probabilities of stem cell division become constant for cells that have acquired both D and R mutations, equating to some self-reliance in growth signals and evasion of apoptosis.

To determine the constant probabilities of symmetric self-renewal, asymmetric self-renewal, and symmetric commitment differentiation, the functional forms for 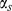, 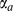, and 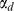 are evaluated at the initial starting time, using mutated parameter values. For example, at 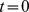, the probability of symmetric self-renewal in cancer stem cells is 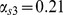, when using the mutated parameter value 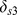. Likewise, the probability of symmetric differentiation in cancer stem cells would initially be 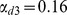. With the probability of apoptosis as 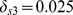 in D-mutated cells, the remainder of the divisions are asymmetric, giving a probability of 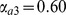. Thus, there is a slight imbalance in symmetric divisions and death of stem cells, causing exponential growth of cancer cells that is maintained over time since feedback mechanisms are not in place to decrease symmetric self-renewal or increase symmetric commitment differentiation. As the cancer population grows without bound, the total stem cell population can surpass the size of the niche because cancer cells do not have this restraint. It is assumed that when the total stem cell population exceeds the niche size, the probability of symmetric self-renewal in regulated cells is zero, and the probability of symmetric differentiation is determined by 
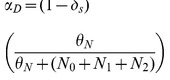
 to ensure that the probabilities of stem cell division are contained between zero and one. In other words, the influence of the stem cell niche on division pattern is removed in cells with both the D and R mutations.

**Figure 6:**
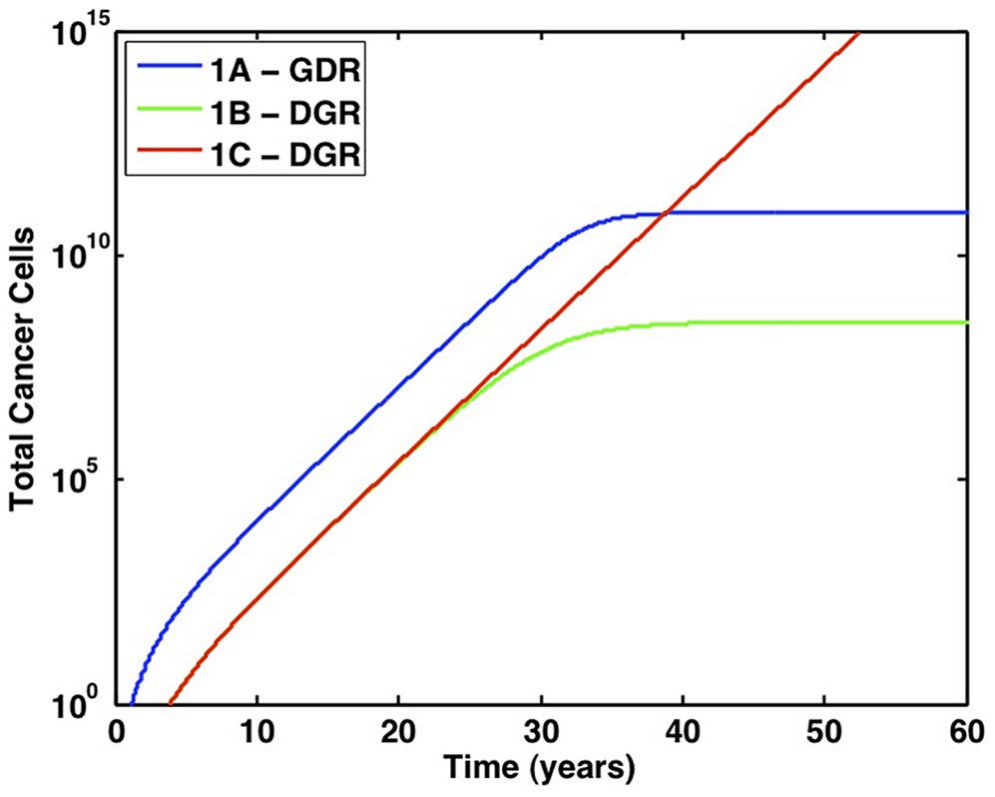
Comparison of fastest pathways for all cases in which stem cell proliferation is increased. GDR is the fastest pathway in Case A, in which all mutations are advantageous (blue). DGR is the fastest pathway in Case B, in which the G and R mutations are lethal in the absence of the D mutation (green). DGR is also the fastest in Case C, in which the loss of regulatory mechanisms causes cancer stem cells to grow exponentially (red).

When the D and R mutations enable unrestricted growth, the initial stages of tumorigenesis do not greatly differ from those determined in the previous section for lethal mutations; however over time, the differences between these cases are noteworthy. Figure 6 plots the total number of cancer cells that result from the fastest pathways. Let Case A be the case in which all mutations are advantageous, Case B represents lethal mutations, and Case C is for unregulated division described in this section. Recall the GDR pathway was fastest when all mutations were advantageous, while the DGR pathway was fastest if lethal mutations were considered. When cancer cells are independent of regulation, the DGR pathway is fastest, and the first cancer stem cell is formed merely 0.3 years faster than when regulation is maintained. However, unlike previous cases, the cancer population not only displaces non-mutated cells, but continues to expand and eventually overtakes the number of cancer cells in both other cases.

The loss of governing mechanisms is not mandatory for the emergence of cancer stem cells, as confirmed by the results in the previous sections on advantageous and lethal mutations. Cancer cells dominate tissues in which homeostatic regulatory mechanisms remain intact due to an acquired competitive advantage over healthy cells. In such circumstances, disease can result from the elimination of healthy cells that have been replaced with mutated cells that do not function properly. However, when mutated cells are also independent of regulation, not only do cancer cells displace non-mutated cells, but exponential growth causes the cancer population to expand uncontrollably. These results imply that tumors composed of cells that have lost tissue-governing mechanisms are more malignant than tumors in which some semblance of regulation is maintained.

### Unbalanced Stem Cell Division

It has been suggested that unbalanced symmetric self-renewal divisions in stem cells may contribute to certain forms of cancer [21], [22], [57], [58]. For instance, the Wnt/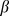-catenin signaling pathway that is important in stem cell self-renewal has also been implicated in cancer [58]. Furthermore, mutations that increase the probability of symmetric self-renewal may even cause more aggressive forms of disease than those that merely increase proliferation. For example, consider Chronic and Acute Myelogeous Leukemia (CML and AML). Patients with CML express BCR-ABL, which increases proliferation, whereas AML patients express NUP98-HOXA9, which increases self-renewal in hematopoietic stem cells and causes a more malignant form of leukemia [7].

Clearly, the mechanisms that govern stem cell self-renewal are of great interest when investigating the emergence of cancer stem cells. With our model that includes regulatory feedback mechanisms, it is possible to examine the impact of mutations that affect stem-cell division properties. Specifically, alteration of 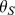 and 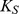, will be addressed since these two parameters determine the probability of symmetric self-renewal. In this section, the R mutation will increase one of these parameters, thereby increasing symmetric self-renewal, while the rate of proliferation will remain unaltered. As before, the D mutation decreases death and the G mutation increases genetic instability.

#### All Mutations are Advantageous

Again consider the case in which all mutations are advantageous. That is, cell death does not increase in mutated cells, and mutated cells have competitive advantage over non-mutated cells. The D mutation decreases the probability of stem cell death and the differentiated cell death rate by half. The G mutation increases the probability at which mutations are acquired from 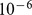 to 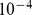. The R mutation doubles the value of 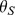, which increases the initial probability of symmetric self-renewal by approximately 10%.

In correlation with the conclusions in the previous section on advantageous mutations that affect proliferation rate, the sooner genetic instability is acquired, the faster cancer stem cells are formed. However, increasing symmetric self-renewal through the doubling of the parameter 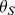 significantly decreases the time to the first cancer stem cell in all pathways. The fastest pathway is GRD, with the first cancer stem cell formed in 8.4 years, nearly eleven years earlier than the appearance of the first cancer stem cell when the R mutation simply leads to an increase in the proliferation rate. Genetic instability is less significant when symmetric self-renewal mutations are considered than when the rate of proliferation increases, as all pathways develop cancer stem cells quickly.

**Figure 7:**
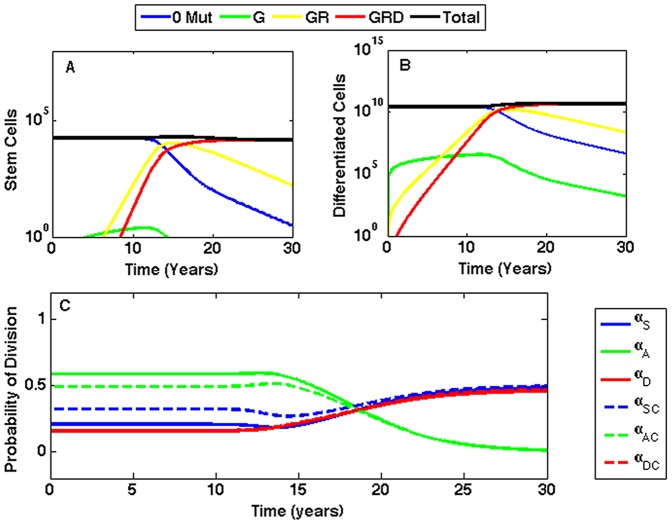
Growth dynamics for the fastest pathway when the R mutation increases symmetric self-renewal. When all mutations are advantageous and the R mutation increases symmetric self-renewal, the GRD pathway is fastest. Cells with the G mutation have probability of mutation 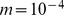; cells with the R mutation have increased half-saturation constant, 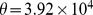 cells; cells with the D mutation have stem cell death rate 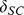 = 0.025/day and differentiated cell death rate 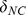 = 1.2/day. (A) Stem cells versus time. The first cancer stem cell is formed in 8.44 years. (B) Differentiated cells versus time. (C) The probabilities for each type of stem cell division versus time. Probabilities for non-mutated cells are denoted 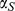, 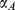, and 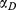, and are plotted with solid lines; probabilities for cancer cells are denoted 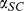, 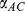, and 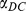, and are plotted with dashed lines.

The growth dynamics of the stem and differentiated cell populations for the GRD pathway are plotted in Figure 7A–B. As illustrated in Figure 7C, the probabilities of stem cell division shift over time to favor symmetric divisions. Doubling 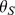 increases the initial probability of symmetric self-renewal from 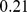 to 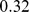, which consequently decreases the probability of asymmetric division. As cancer cells displace non-mutated cells, the Hill functions in both symmetric self-renewal and symmetric commitment differentiation tend to one. In both non-mutated and cancer stem cells, the probability of symmetric self-renewal goes to 0.5, the probability of symmetric commitment differentiation goes to 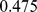, and the probability of asymmetric division goes to zero.

An imbalance in favor of symmetric self-renewal causes rapid expansion in mutated cells and quickly displaces healthy cells. In approximately 15 years, cancer dominates the tissue. Although the probability of symmetric self-renewal is initially increased in cells with the R mutation, regulatory mechanisms were not completely eliminated. As a result, the initial rapid expansion is eventually controlled, preventing unrestricted tissue growth. This implies that altered regulations may still be capable of mediating homeostasis, even if it is abnormally controlled.

#### Lethal Mutations

Now suppose suppose that G and R mutations are not advantageous in cells that have not previously acquired the D mutation. In stem cells that have G and/or R but not D, the probability of death is 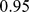. Under these assumptions, the DGR is the fastest pathway, but the first cancer stem cell forms in 11.7 years, less than half of the time required by the same pathway when the proliferation rate is altered rather than symmetric self-renewal. In fact, a cancer stem cell is formed in all pathways within 18 years. This is remarkable because the slowest pathway initiates cancer before even the fastest pathway did in the previous sections where the proliferation rate was increased.

**Figure 8:**
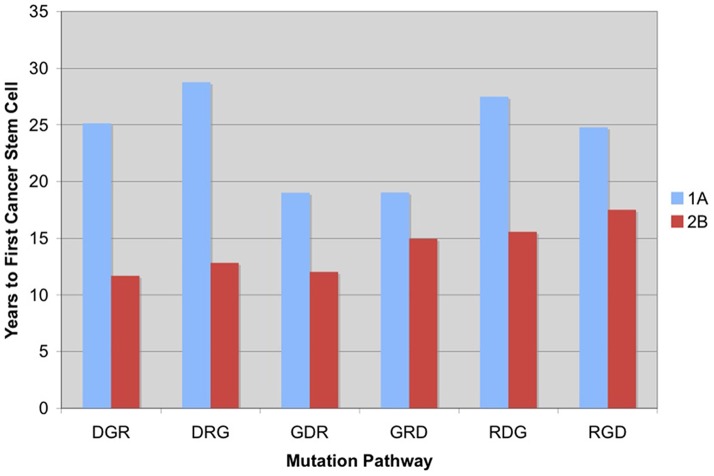
Increased symmetric self-renewal speeds cancer onset more than increased proliferation rate. The time to first cancer stem cell is faster for increased symmetric self-renewal when all mutations are not advantageous (Case 2B) even when compared to the case where all mutations are advantageous with increased proliferation rate (Case 1A).

Figure 8 compares the time to first cancer stem cell for each pathway when all mutations are advantageous and R increases the proliferation rate versus those in which R increases symmetric self-renewal and G and R are lethal. Increasing symmetric self-renewal approximately 10% by doubling 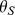 dramatically decreases the time to first cancer stem cell in comparison with increasing the rate of stem-cell proliferation. It is therefore suggested that increasing unbalanced symmetric divisions causes malignancies to develop quicker than increasing the rate of division. Furthermore, symmetric self-renewal minimizes the differences in cancer initiation when comparing all pathways. As a result, unbalanced symmetric self-renewal dictates a faster pace of cancer development, regardless of the sequential order of mutations.

#### Mutations Affecting Regulatory Mechanisms

The last two cases demonstrate that cancer cells can emerge from increased symmetric self-renewal, even if regulatory mechanisms are not completely lost. Based on the conclusions that unbalanced symmetric divisions speed the onset of cancer more than increased stem cell proliferation, one would predict that unregulated symmetric divisions would be additionally problematic. Indeed, if stem cells with both the R and D mutations become independent of division regulation and regulatory mechanisms are lost, the cancer stem cell population emerges quickly and grows exponentially.

**Figure 9:**
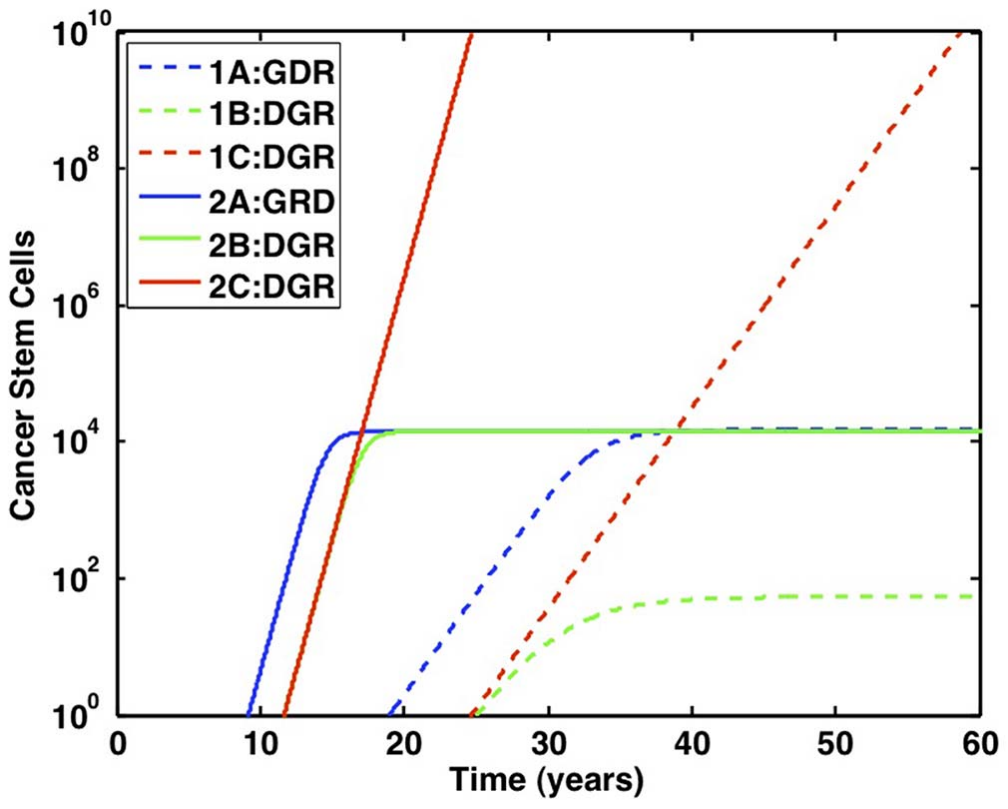
Complete loss of regulation enables malignant growth. Case 1 simulations, in which stem cell proliferation is increased (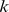 = 0.07/day), are plotted with dashed lines. Case 2 simulations, in which symmetric self-renewal is increased (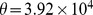 cells), are plotted with solid lines. For each case, A denotes when all mutations are advantageous, B denotes lethal mutations, and C denotes the loss of regulatory mechanisms. The first cancer stem cell is formed via the GDR pathway when symmetric self-renewal is increased and all mutations are advantageous. The most malignant growth is formed through the DGR pathway, when stem cells have increased symmetric self-renewal and have also lost feedback regulation.

To emphasize the significance increasing symmetric self-renewal has on cancer stem cell dynamics, the cancer stem cell population of the fastest pathways from each of the six cases discussed thus far are plotted in Figure 9. Case 1 denotes all the simulations in which the R mutation doubles the proliferation rate of stem cells, while Case 2 denotes the simulations in which the R mutation doubles 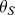 and increases symmetric self-renewal. All mutations are advantageous in Cases 1A and 2A, G and R mutations are not advantageous without D in Cases 1B and 2B, and stem-cell division regulation is lost in cells with D and R mutations in Cases 1C and 2C.

The fastest pathways in which R increases symmetric self-renewal are significantly faster than the pathways in which R increases the stem-cell proliferation rate. In addition, when comparing Cases 1C and 2C in which the cancer stem-cell population grows exponentially, the rate at which cancer grows is markedly increased in the latter case. Therefore, deregulated unbalanced symmetric self-renewal quickly initiates tumorigenesis and continues to promote cancer expansion through an elevated growth rate if regulatory mechanisms are lost.

#### Mutations Affecting the Stem Cell Niche

It has been suggested that cancer stem cells are not as dependent on the stem cell niche as normal stem cells [16], [15]. As a result, cancer stem cells are not as restricted by the physical carrying capacity of the niche. In the previous sections, all control from the stem cell niche was removed, and cancer stem cells grew exponentially. Now a case is considered in which cancer stem cells are still restricted by the niche, but the niche controlling mutated cells is larger since it is assumed mutated cells have more freedom in where they reside. It is likely that increasing the niche will have a major impact on tumor growth. In the following simulations, the R mutation doubles the size of the stem cell niche, 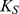, but does not change the proliferation rate, 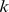, or 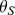. As before, the D mutation decreases the death rate, the G mutation increases the mutation rate, and it is assumed that the G and R mutations are not advantageous unless D has been acquired.

The results of the previous section demonstrated that increasing 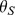 increases symmetric self-renewal, but now doubling 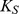 causes an even greater increase in symmetric self-renewal divisions. In addition, increasing 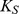 also decreases the probability of symmetric commitment differentiation. The combination of the increase in symmetric self-renewal and decrease in symmetric commitment differentiation creates an even larger imbalance of symmetric divisions than doubling 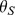. Consequently, it is not surprising that a mutation increasing the stem cell niche causes the fastest cancer onset. The DGR pathway is fastest, forming the first cancer stem cell in 6.65 years, though all pathways have a cancer stem cell in under ten years.

There is an additional interesting aspect of the growth dynamics caused by this mutation. The increased niche capacity for R-mutated stem cells give them a significant competitive advantage other cells. As mutated cells fill up the niche, feedback regulation forces symmetric self-renewal of normal cells to go to zero since the system does not want to add more stem cells. In addition, symmetric commitment differentiation of normal cells goes to its maximum value of 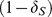 in order to push cells out of the niche. In so doing, the normal cell population differentiates more than it self-renews, which in turn causes the forced rapid extinction of normal cells.

**Figure 10:**
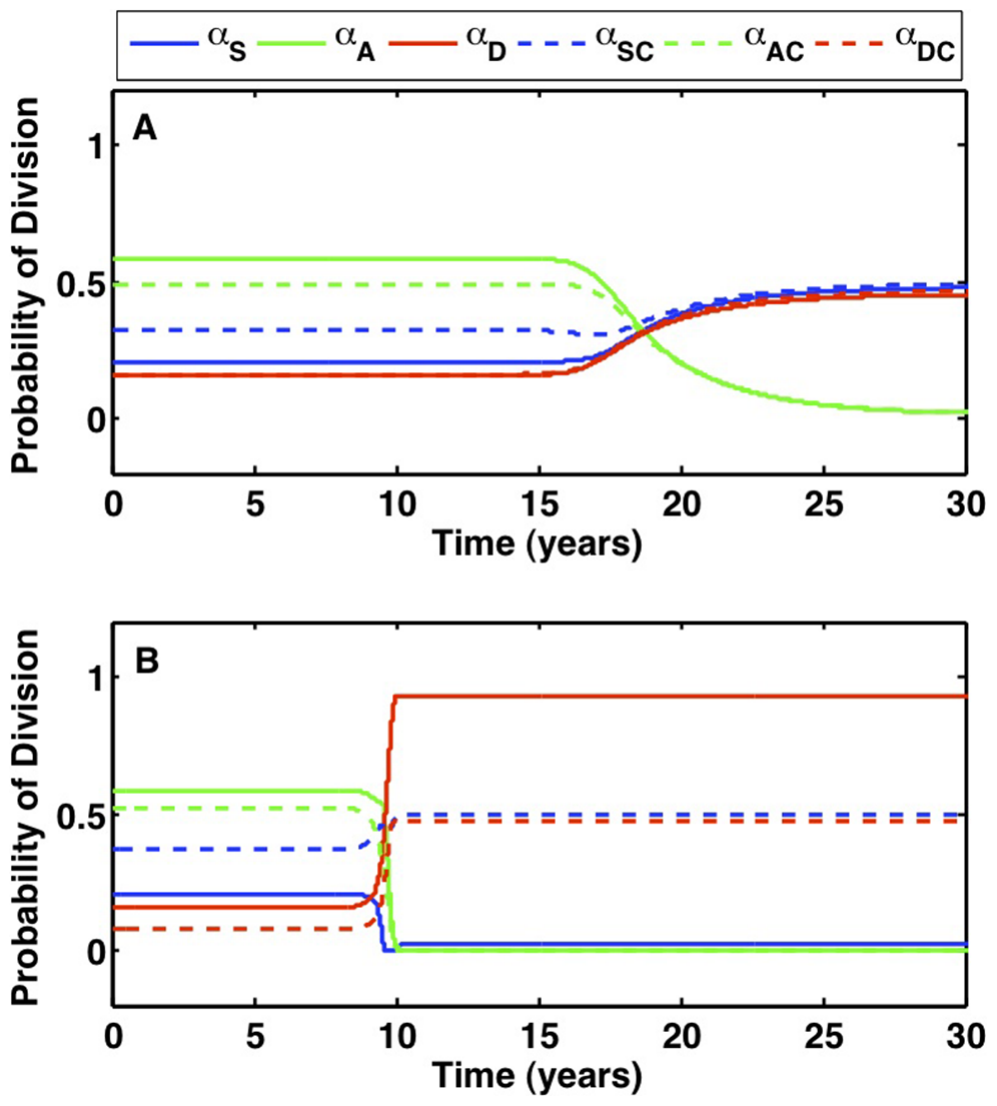
Comparison of stem cell division probabilities for mutations that increase symmetric self-renewal. The fastest pathway of both cases is DGR, but the probabilities of stem-cell division are markedly different. Values for non-mutated cells are plotted with solid lines, cancer cells are plotted with dashed lines. (A) The probabilities of stem cell division when the R mutation doubles the half-saturation constant, such that 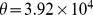 cells. Both mutated and healthy cells approach balanced division patterns in the long run. (B) The probabilities of stem cell division when the R mutation doubles the niche size, such that 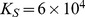. Normal cells are forced to differentiate due to crowding from the niche.

Figure 10 compares the probabilities of stem cell division that occur in a system following the DGR pathway for when symmetric self-renewal increases due to increasing 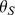, as opposed to when it increases because of the niche, 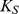. When symmetric self-renewal is increased by doubling 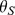 all cells continue to be regulated by the same size niche. As time progresses, non-cancer cells diminish, which forces the chemical signaling term 
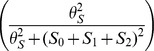
 to one and symmetric self-renewal in both normal and cancer cells goes to 50%, as demonstrated in Figure 10A. The other 50% of divisions result in symmetric commitment differentiation and apoptosis. In contrast, when the R mutation doubles the niche, the signaling term also goes to one as time progresses, but because cancer cells fill up the niche and can in fact surpass the niche, symmetric self-renewal in normal cells diminishes while symmetric commitment differentiation goes to 95%. Cancer cells do not have the same division probabilities as normal stem cells in the long run due to the increased niche capacity; cancer stem cells symmetrically self-renew at 50% and symmetrically differentiate at 47.5%, as plotted in Figure 10B. In summary, a mutation that increases the cells self-reliance apart from the niche, thereby increasing the potential niche capacity in which the cell may reside, creates a considerable imbalance in stem cell division probabilities while also promoting extensive differentiation and loss of normal cells.

## Discussion

Although many types of mutations have been identified in cancer cells, it is difficult to determine the order in which they were acquired that led to malignancy. This paper focuses on investigating mutation acquisition in hierarchical tissue in which stem cell division is governed by regulatory mechanisms that promote homeostasis. In particular, the sequential accumulation of somatic mutations was modeled to examine the multi-step process that initiates cancer. For each mutation pathway considered, the time to first cancer stem cell determined the onset of malignancy, so that the fastest pathway could be established. Importantly, the model predicts that the order in which mutations are acquired significantly affects the pace of tumorigenesis. In addition, tumor composition varies for different mutation pathways, so that some sequences generate tumors that are dominated by cancerous cells, while others are primarily comprised of cells with only one or two mutations.

In addition to the importance of the sequence of mutations, model predictions indicate that certain types of mutations are more significant than others in dictating cancer onset. We specifically investigated mutations that increase rate of stem cell division without affecting the type daughter cell produced and mutations that increase probability of stem cell symmetric self-renewal without affecting the rate of division. In both cases, the model predicts that when all mutations are advantageous, genetic instability is the key determining factor for the emergence of cancer stem cells, and this result is robust for a wide range of parameters. Although pathways beginning with genetic instability are fastest for both types of R mutations, its effects are significantly diminished when symmetric self-renewal is increased, demonstrating that aberrant symmetric self-renewal may instigate aggressive malignancies. Over all, mutations that disturb the balance between symmetric self-renewal and differentiation initiate cancer faster than mutations that simply increase the stem cell proliferation rate. The result that the fastest pathways acquired genetic instability first agrees with the results of Michor et al., who predicted that chromosomal instability was an early event in colon cancer [32]. This result differs from the work by Spencer et al., who predicted that the fastest pathway to cancer ends with genetic instability. Rather than following the particular order in which mutations accumulate, however, Spencer et al. [31], did not distinguish the chronological order of mutations that generated cells with a particular phenotype. In contrast, the predictions presented here suggest that the specific sequential order of mutation acquisition decisively influences tumor dynamics. Particularly significant are mutations that cause the stem cell division pattern to be unbalanced in the favor of symmetric self-renewal. Increased symmetric self-renewal significantly quickens cancer onset and progression because it rapidly expands the cancer stem cell population. Furthermore, it diminishes the importance of all other mutations, including genetic instability, in that cancer stem cells emerge in all pathways within a relatively short time of each other.

Our model predictions are in line with other models of mutation acquisition in the literature [39], [36]; however, there are many aspects of tumor dynamics that we can now investigate to a greater extent with the incorporation of regulatory mechanisms, which are missing from many published approaches. For instance, strong evidence has emerged that links stem cell fate to their proximity to specialized domains. This has led to the concept of the stem cell niche, specific anatomic locations that regulate how stem cells participate in tissue generation, maintenance, and repair [44], [5]. Assuming that the amount of space in such microenvironments (or niches) is limited, the number of stem cells is also limited by the number that can fit in that space. This suggests an environmental carrying capacity for the stem cell population, which is a completely different mechanism of regulation that we have included in our model. It has also been suggested [5] that the relation between stem cells and the niche is likely to be a symbiotic one in which stem cells may be capable of both regulating and regenerating the niche. This implies that stem cells themselves help regulate their own proliferation and self-renewal capacity. In fact, the Hill function we use in (11) reflects autocrine effects on stem cell self-renewal by accounting for the situation where if the number of stem cells, S, approaches zero, the probability of symmetric self-renewal based on chemical signaling in the niche approaches the maximum value. With the inclusion of feedback mechanisms like these that govern stem cell division, cancer cells do not necessarily grow exponentially as seen in [39]. Rather, if regulation remains intact, even though it may be abnormal, a new equilibrium is achieved. Furthermore, unlike the predictions in [39], healthy cells diminish due to the displacement by mutated cells that have a competitive advantage in the niche. Finally, in the event that all regulation is lost in cancer cells, exponential growth of the cancer population occurs in addition to the depletion of normal cells. Therefore, the model predicts that stem cell regulatory mechanisms maintain system homeostasis under healthy conditions and cancer is easily initiated when they are lost. Consequently, feedback regulation controlling stem cell self-renewal and differentiation can prevent exponential growth despite any alterations in normal tissue growth, but complete loss of this regulation initiates unrestricted expansion in cancerous populations.

Recently, Rodgriguez-Brenes et al., also found that the sequence in which mutations are acquired is crucial for determining the growth dynamics of tumors. Also, in agreement with the results presented here, they found that regulation of the balance between differentiation and stem cell self-renewal is a more critical mediator of the overall growth pattern than the rate of cell proliferation. There is also agreement in the consequences for the partial and total loss of feedback. However, the types of regulatory mechanisms considered in [59] differ from those discussed here, as do many of the questions explored and biological implications highlighted. The mathematical model presented here provides a general framework that can be used to investigate tumorigenesis in any hierarchical tissue. To demonstrate the usefulness of this model, simulations of mutation acquisition with parameters taken from the hematopoietic cells were conducted, but the model structure is general enough to be adapted to other tissues and include any number of mutations. Future modifications of the model will include explicitly adding in the progenitor cell pool and studying a variety of stem cell targeted treatment strategies.

## Supporting Information

Table S1 **Increased Stem Cell Proliferation Parameters.**
(PDF)

Table S2 **Unbalanced Stem Cell Division Parameters.**
(PDF)
