# Supplementary material for: A Mathematical Model of Cancer Stem Cell Driven Tumor Initiation: Implications of Niche Size and Loss of Homeostatic Regulatory Mechanisms
Source: PLoS One. 2013 Aug 19;8(8):e71128. doi: 10.1371/journal.pone.0071128 (PMC3747196; doi:10.1371/journal.pone.0071128)
Supplement: Table S2 — Unbalanced Stem Cell Division Parameters. (PDF) [file pone.0071128.s002.pdf]

Table S2: Unbalanced Stem Cell Division Parameters

	GRD		DGR		RDG	
Parameter	AM	LM	AM	LM	AM	LM
m_0	10^{-6}	10^{-6}	10^{-6}	10^{-6}	10^{-6}	10^{-6}
θ_{S0}	$1.96x10^4$	$1.96x10^4$	$1.96x10^4$	$1.96x10^4$	$1.96x10^4$	$1.96x10^4$
δ_{S0}	0.05	0.05	0.05	0.05	0.05	0.05
δ_{N0}	2.4	2.4	2.4	2.4	2.4	2.4
m_1	10^{-4}	10^{-4}	10^{-6}	10^{-6}	10^{-6}	10^{-6}
θ_{S1}	$1.96x10^4$	$1.96x10^4$	$1.96x10^4$	$1.96x10^4$	$3.92x10^4$	$3.92x10^4$
δ_{S1}	0.05	0.95	0.025	0.025	0.05	0.95
δ_{N1}	2.4	2.4	1.2	1.2	2.4	2.4
m_2	10^{-4}	10^{-4}	10^{-4}	10^{-4}	10^{-6}	10^{-6}
θ_{S2}	$3.92x10^4$	$3.92x10^4$	$1.96x10^4$	$1.96x10^4$	$3.92x10^4$	$3.92x10^4$
δ_{S2}	0.05	0.95	0.025	0.025	0.025	0.025
δ_{N2}	2.4	2.4	2.4	2.4	1.2	1.2
θ_{S3}	$3.92x10^4$	$3.92x10^4$	$3.92x10^4$	$3.92x10^4$	$3.92x10^4$	$3.92x10^4$
δ_{S3}	0.025	0.025	0.025	0.025	0.025	0.025
δ_{N3}	1.2	1.2	1.2	1.2	1.2	1.2

AM = Advantageous Mutations, LM = Lethal Mutations
